# Distinct evolutionary patterns of *Oryza glaberrima* deciphered by genome sequencing and comparative analysis

**DOI:** 10.1111/j.1365-313X.2011.04539.x

**Published:** 2011-03-21

**Authors:** Hiroaki Sakai, Hiroshi Ikawa, Tsuyoshi Tanaka, Hisataka Numa, Hiroshi Minami, Masaki Fujisawa, Michie Shibata, Kanako Kurita, Ari Kikuta, Masao Hamada, Hiroyuki Kanamori, Nobukazu Namiki, Jianzhong Wu, Takeshi Itoh, Takashi Matsumoto, Takuji Sasaki

**Affiliations:** 1 Division of Genome and Biodiversity Research, National Institute of Agrobiological SciencesTsukuba, Ibaraki 305-8602, Japan; 2 Research Division I, Institute of the Society for Techno-innovation of Agriculture, Forestry and FisheriesTsukuba, Ibaraki 305-0854, Japan; 3 Tsukuba Division, Mitsubishi Space Software Co., Ltd.Tsukuba, Ibaraki 305-0032, Japan

**Keywords:** *Oryza glaberrima*, African rice, genome sequencing, genome evolution

## Abstract

Here we present the genomic sequence of the African cultivated rice, *Oryza glaberrima*, and compare these data with the genome sequence of Asian cultivated rice, *Oryza sativa*. We obtained gene-enriched sequences of *O. glaberrima* that correspond to about 25% of the gene regions of the *O. sativa* (*japonica*) genome by methylation filtration and subtractive hybridization of repetitive sequences. While patterns of amino acid changes did not differ between the two species in terms of the biochemical properties, genes of *O. glaberrima* generally showed a larger synonymous–nonsynonymous substitution ratio, suggesting that *O. glaberrima* has undergone a genome-wide relaxation of purifying selection. We further investigated nucleotide substitutions around splice sites and found that eight genes of *O. sativa* experienced changes at splice sites after the divergence from *O. glaberrima*. These changes produced novel introns that partially truncated functional domains, suggesting that these newly emerged introns affect gene function. We also identified 2451 simple sequence repeats (SSRs) from the genomes of *O. glaberrima* and *O. sativa*. Although tri-nucleotide repeats were most common among the SSRs and were overrepresented in the protein-coding sequences, we found that selection against indels of tri-nucleotide repeats was relatively weak in both African and Asian rice. Our genome-wide sequencing of *O. glaberrima* and in-depth analyses provide rice researchers not only with useful genomic resources for future breeding but also with new insights into the genomic evolution of the African and Asian rice species.

## Introduction

As the demand for food increases, genomic approaches are anticipated to expedite the creation of improved cultivars of important cereal crops such as rice. Here we provide genome sequence information for an African rice species. In the genus *Oryza*, there are two cultivated rice species, *Oryza glaberrima* (*Og*) and *Oryza sativa* (*Os*), which diverged about 640 000 years ago (Ma and Bennetzen, 2004). While *Os* is distributed and cultivated in diverse areas of the world, *Og* is endemic to Africa. Genome sequences of *Os* (Goff *et al.*, 2002; International Rice Genome Sequencing Project, 2005; Yu *et al.*, 2005; Itoh *et al.*, 2007) have contributed greatly to the identification of agronomically useful genes and loci (Izawa *et al.*, 2009). Thus, a genome sequence from *Og* should also add to knowledge of not only African rice but also other *Oryza* species in general because comparative genome sequence analyses deepen our understanding of characteristics of related species at the molecular level (Paterson *et al.*, 2009). In this study, we present gene-enriched genomic sequences of *Og* that were determined by the whole genome shotgun method and also show results of comparative analyses with the *Os* genome.

While *Os* was domesticated about 10 000 years ago (Khush, 1997), *Og* may have a shorter history; it was derived from its wild ancestor, *Oryza barthii*, about 2000–3500 years ago (Sarla and Swamy, 2005). A number of differences in morphological and ecological characteristics between *Og* and *Os* indicates that the domestication process for *Og* was less intense than that for *Os* in terms of high grain yield, enhanced palatability, and other desirable characteristics (Sarla and Swamy, 2005). In addition to these traits, *Og* has other advantageous traits, such as strong weed competitiveness and tolerance to drought, salinity, pests, and diseases. Because *Og* offers traits of potential value to rice breeding programs, the Africa Rice Center (formerly known as the West Africa Rice Development Association) started, in 1992, to develop interspecific hybrids between *Os* and *Og* in an attempt to combine the superior features of both species. Despite the strong reproductive barriers between *Os* and *Og*, the effort to construct progeny was successful in the mid-1990s (Jones *et al.*, 1997). The new cultivar, NERICA (New Rice of Africa), possessed favorable hybrid traits such as high yield obtained from *Os* and high weed competitiveness, drought tolerance, and pest or disease resistance derived from *Og*. In addition to improvement of *Og* by *Os* genes, *Og* is also expected to be a useful genetic resource that could improve Asian cultivated rice in terms of grain quality and resistance to biotic stresses (Aluko *et al.*, 2004; Li *et al.*, 2004; Gutierrez *et al.*, 2010). Therefore, knowledge of the genome sequence of *Og* should facilitate research toward genomic breeding of both African and Asian rice species. Furthermore, the genome sequence of *Og* is expected to help researchers understand the evolution of rice species.

To open the door to a new genomics era of *Oryza* species, we present genome sequence analyses of *O. glaberrima* (IRGC104038), a cultivar that has characteristics typical of *Og* (Doi *et al.*, 1998). Comparing the *Og* genome sequence with those of *japonica* (*Osj*) and *indica* (*Osi*) cultivars of *Os*, we seek to answer three questions. First, how different are nucleotide substitutions, gene contents, and selective constraints acting on the genes between *Og* and *Os*? Second, how much did substitutions that occurred after the speciation of *Og* and *Os* affect the gene structures and functions? Third, is selection against indels of simple sequence repeats (SSRs), which are widely used to analyze genetic diversity, develop molecular markers, and investigate gene and genome evolution (Metzgar *et al.*, 2000; McCouch *et al.*, 2002; Morgante *et al.*, 2002; Garris *et al.*, 2005), different among species? Furthermore, the importance of *Og* as a genetic resource is discussed.

## Results and Discussion

### *Oryza glaberrima* genome sequence as a resource for breeding based on genomics

Our whole-genome shotgun sequencing yielded a total of 206 317 153 bp of 437 642 sequence reads, which corresponds to about 0.6× coverage of 357 Mbp of the *Og* genome (Kim *et al.*, 2008). Repeat elements accounted for 22.5% of the total nucleotides, which was significantly smaller than 29% observed in a previous study (*G*-test, *P* < 1.0 × 10^−15^) (Ammiraju *et al.*, 2006) (Table 1). This observation is due to the fact that we employed methods of subtractive hybridization and methylation filtration. In particular, the latter method can capture low-methylated genomic regions and discard methylated regions that are mainly composed of repetitive elements (Whitelaw *et al.*, 2003). In fact, the fraction of repetitive elements detected in the sequences derived by methylation filtration was as low as 10%, indicating that this method is effective in capturing non-repetitive genomic portions of the *Og* genome (Table 1). However, repetitive elements accounted for a relatively large fraction of the sequences derived by subtractive hybridization. To precisely examine the efficacy of the subtractive hybridization, we searched the subset derived by subtractive hybridization for the target repetitive elements by BLASTN (see Experimental procedures). As a result, it was found that our subset contained significantly smaller fraction of the target repetitive elements (*G*-test, *P* < 1.0 × 10^−15^) than a non-filtered sequence set previously studied (Ammiraju *et al.*, 2006) (Table S1). This result indicates that our subtractive hybridization could effectively screen target repetitive elements out.

**Table 1:** Statistics of the *Oryza glaberrima* genome sequence

	Total	Methylation filtration	Subtractive hybridization
Total number of sequences	437 642	219 203	218 439
Total length of sequences (bp)	206 317 153	107 419 702	98 897 451
Fraction of repetitive elements (%)	22.5	10.4	35.0
Number of filtered sequences	368 537	205 601	162 936
Total length of filtered sequences (bp)	175 584 862	101 381 318	74 203 544
Number of mapped sequences	256 801	156 909	99 892
Coverage of the *Oj* genome (bp)	69 083 576	45 795 595	30 226 496

*Oj*, *Oryza sativa japonica*.

**Figure 1:**
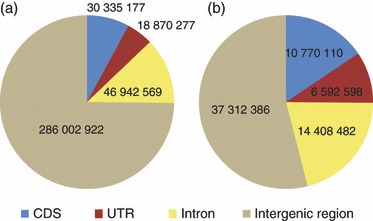
Compositions (bp) of sequence types (a) in the total *Oryza sativa japonica* (*Osj*) genome and (b) in the *Osj* genomic regions covered by *Oryza glaberrima* sequences.

Highly repetitive shotgun sequence reads were removed from the dataset during the repeat-masking process. In total, 256 801 (69.7%) of 368 537 non-repetitive sequence reads were successfully mapped to the *Osj* genome. The mapped reads were concatenated on the reference genome, resulting in 148 435 contigs covering 69 083 576 bp (approximately 18%) of the *Osj* genome (Table 1). The *Og* contigs were expected to be enriched for gene regions because of the repeat subtraction and methylation filtration processes. Our comparison of *Og* contigs with the *Osj* genomic regions annotated by the Rice Annotation Project (RAP) (Tanaka *et al.*, 2008) proved this to be the case (Figure 1). The *Og* sequences covered 10 080 *Osj* genes with length ≥100 amino acids or >70% coverage. Furthermore, 25.1% of the *Og* nucleotides mapped on the *Osj* genome corresponded to *Osj* exons found by the RAP. These results indicate that the gene sequences of *Og* were efficiently captured in our sequence set.

**Figure 2:**
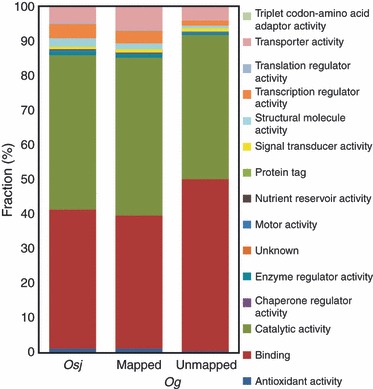
Functional classification of *Oryza sativa japonica* (*Osj*) and *Oryza glaberrima* (*Og*) proteins. The classifications of mapped and unmapped sequences of *Og* were derived from the Swiss-Prot database proteins that were homologous to the mapped and unmapped sequences (see Experimental procedures). Protein categories are based on the molecular functions of the Gene Ontology hierarchy.

Genomic sequences deleted in the *Osj* lineage were previously reported to contain a significant number of agronomically useful genes (Sakai and Itoh, 2010). To assess the types of gene that resided in the *Og*-specific genomic portions, we first assembled unmapped reads to eliminate redundancies. After producing contigs by the CAP3 program, the unmapped reads consisted of 4598 contigs and 20 461 singletons, consisting of 12 728 728 bp in total (Table 2). Because these contigs and singletons did not show significant similarity to the *Osj* genome, they were suitable candidates to be *Og*-specific genomic sequences. Similarity searches of the contigs and singletons against the Swiss-Prot database were conducted, and possible functional categories based on the Gene Ontology annotation were assigned (for details, see Experimental procedures). Likewise, functions of mapped contigs were inferred (Figure 2). Although the functional classification of mapped contigs showed a similar pattern to that for the *Osj* genes (Figure 2), in the unmapped (*Og*-specific) sequences, the nucleotide-binding (NB-ARC) and leucine-rich repeat domains, which are generally characteristics of disease-resistant genes, and protein kinase domains were significantly overrepresented (Table 3). It was previously shown that *Osj* had lost a significant number of disease-resistance-related genes that were preserved in wild rice species and *Og* (Sakai and Itoh, 2010). Because the accession number (IRGC104038) of *Og* used in this study is different from that (IRGC96717) of Sakai and Itoh (2010), it is probable that unique disease-resistance-related genes missing in *Os* are widely preserved in the diverse accessions of *Og*. Genomes of landraces and wild rice species are expected to harbor important genes that enhance yield, resistance to disease and insects, and various abiotic resistances (Brar and Khush, 1997; Kovach and McCouch, 2008). In fact, novel alleles that improve grain quality have been identified in *Og* (Aluko *et al.*, 2004; Li *et al.*, 2004). Our results shed light on the potential of *Og* as a useful genetic resource for the future development of new cultivars.

**Table 2:** Statistics of mapped and unmapped reads

No. of mapped sequences	256 801
No. of mapped contigs	148 435
N50 of the mapped contigs (bp)	603
No. of unmapped sequences	111 736
No. of unmapped contigs	4598
No. of unmapped singlets	20 461
Total length of contigs and singlets (bp)	12 728 728
Average length of the unmapped contigs (bp)	766
Average length of the unmapped singlets (bp)	450

**Table 3:** The 10 most frequent domains among the unmapped sequences of *Oryza glaberrima*

		Mapped		Unmapped		
				
InterPro ID	Description	No. of genes with the domain	No. of genes without the domain	No. of genes with the domain	No. of genes without the domain	*P*-value
IPR000719	Protein kinase, catalytic domain	377	3364	85	446	9.14 × 10^−5^
IPR001611	Leucine-rich repeat	111	3630	82	449	2.20 × 10^−16^
IPR002182	NB-ARC	50	3691	33	498	1.99 × 10^−10^
IPR003591	Leucine-rich repeat, typical subtype	29	3712	20	511	5.01 × 10^−7^
IPR008271	Serine/threonine-protein kinase, active site	322	3419	70	461	1.13 × 10^−3^
IPR011009	Protein kinase-like domain	387	3354	91	440	1.10 × 10^−5^
IPR013210	Leucine-rich repeat-containing N-terminal domain, type 2	52	3689	40	491	5.73 × 10^−14^
IPR016040	NAD(P)-binding domain	188	3553	32	499	3.4 × 10^−1^
IPR017441	Protein kinase, ATP binding site	343	3398	78	453	1.52 × 10^−4^
IPR017442	Serine/threonine-protein kinase-like domain	355	3386	81	450	9.83 × 10^−5^

The assembled genome sequences presented in this study are accessible through our website (http://green.dna.affrc.go.jp/Og/). All the sequence reads are registered in DDBJ/EMBL/GenBank (accession numbers FT434720–FT872361).

### Biased nucleotide substitutions across genomes

**Figure 3:**
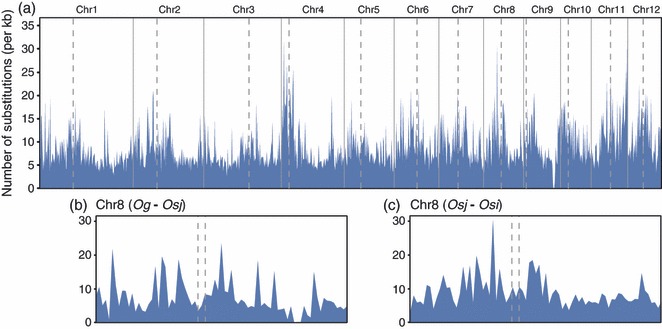
Distribution of the number of nucleotide substitutions. Distribution of the number of nucleotide substitutions between (a) *Oryza glaberrima* (*Og*) and *Oryza sativa japonica* (*Osj*) on the 12 chromosomes, (b) *Og* and *Osj* on chromosome 8, and (c) *Osj* and *Oryza sativa indica* (*Osi*) on chromosome 8. Nucleotide substitutions were counted in 10-kb windows with 10-kb steps along the chromosomes. Dashed lines show approximate positions of the centromeres.

We investigated patterns of nucleotide substitutions in *Og* and *Osj* by aligning the genomes of the two species. The ratios of transitions to transversions ranged from 1.63 to 1.81 with an average of 1.72 across the 12 chromosomes, which was comparable to the ratios in maize and wheat (Bousquet *et al.*, 1992). We then plotted the number of nucleotide substitutions in 10-kb windows across the *Osj* genome (Figure 3). To avoid sequencing errors, we selected only nucleotides supported by two or more shotgun reads with the phred score of ≥20, and if different nucleotides were seen at the same site, one nucleotide that was in the majority was used. Because different selective constraints on exonic regions result in different nucleotide substitution patterns, only non-exonic sequences, in which purifying selection is generally weak, were used to minimize the variation in nucleotide substitution. Nonetheless, biased distributions of the substitutions were observed in all 12 chromosomes. In particular, the differences between the two species were relatively higher around the centromeric regions (Figure 3a).

We examined chromosome 8 in greater detail because 2.18 Mbp of the centromeric and pericentromeric regions has been fully sequenced in *Osj* (Wu *et al.*, 2004). Although regions with high polymorphism were observed around the pericentromeric regions, the level of polymorphism was relatively low inside the centromeric and pericentromeric regions (Figure 3b,c), which is consistent with similar observations in the maize genome (Gore *et al.*, 2009). The reduction of polymorphism in the centromeric and pericentromeric regions might be explained by the suppression of recombination (Harushima *et al.*, 1998).

### Natural selection against amino acid substitutions

To further investigate the genomic evolution of the African and Asian rice species, we examined amino acid substitutions that occurred specifically in *Og* and *Osj*. Based on the codon positions of *Osj* genes retrieved from the Rice Annotation Project Database (RAP-DB) (Tanaka *et al.*, 2008), amino acid alignments of 2161 genes of *Og*, *Osj*, and *Sorghum bicolor* were generated. We used *Sorghum bicolor* as an outgroup so that we could infer lineage-specific substitutions of *Og* and *Osj*.

If natural selection varied between these two species after speciation, then amino acid substitution patterns may differ between their proteins. To assess this hypothesis, we first estimated the number of amino acid substitutions between *Og* and *Osj* that accumulated after speciation using the Poisson correction with a gamma parameter of 2.25. Each branch length was estimated by the least squares method. As a result, the branch lengths leading to *Og* and *Osj* were 2.5 × 10^−3^ and 2.7 × 10^−3^, respectively, and we found no significant difference in the evolutionary rates between the species (Tajima’s relative rate test, *P* = 0.25) (Tajima, 1993). Next, we counted the number of lineage-specific substitutions. In 2067 genes, there were 33 175 amino acid sites in which substitutions were found, and 286 and 315 of them were parsimoniously inferred to be *Og*- and *Osj*-specific substitutions, respectively (Table S2). Then we examined the number of substitutions that do or do not represent changes in amino acid properties. We used four different classifications of amino acid properties (Dayhoff *et al.*, 1978; Zhang, 2000; Hanada *et al.*, 2006, 2007) and tested the statistical significance of changes between *Og* and *Osj* by a *G*-test. No significant difference was found using any of the four classifications. These observations indicate that patterns of amino acid substitutions were essentially the same between the two species in terms of property changes. Therefore, natural selection processes at the amino acid level did not seem to differ significantly.

We further investigated the difference in selective constraints against amino acid substitutions. We calculated the synonymous and nonsynonymous distances in each lineage of *Og* and *Osj* by the modified Nei–Gojobori method (Zhang *et al.*, 1998), and the branch lengths were estimated by the least squares method (Table S3). The ratio of the nonsynonymous to synonymous distances of *Og* (0.30) was significantly larger than that of *Osj* (0.25); the total numbers of nonsynonymous and synonymous substitutions were significantly different between *Og* and *Osj* at the 5% level (*G*-test, *P* = 0.04) (Table S3). This result suggests that *Og* may have experienced genome-wide relaxation of purifying selection. Previous studies of microsatellites have shown that heterozygosity of *Og* was 0.22–0.29 and that that of *Osj* was about 0.62 (Semon *et al.*, 2005; Gao and Innan, 2008); thus, the heterozygosity was smaller in *Og* than in *Osj*. If mutation rates are similar, lower heterozygosity is equivalent to a smaller effective population size (Ota and Kimura, 1973; Kimura, 1983). Hence, *Og* may have a lower effective population size – perhaps due to a more severe domestication bottleneck than in *Osj*. Another possibility is that diverse genetic variations were introduced into *Osj* during intense domestication, which led to apparent acceleration of the evolutionary rate (Gao and Innan, 2008).

### Lineage-specific nucleotide substitutions at splice sites

The emergence of a new splice site in a protein-coding region may result in more drastic phenotypic changes than nucleotide substitutions at other sites because the protein product might be truncated, and the function greatly altered. For example, a mutation at the donor site of the first intron in the rice *waxy* gene causes a reduction in the waxy protein content, leading to a low amylose content in seeds (Wang *et al.*, 1995; Yamanaka *et al.*, 2004). We aligned 52 537 splice sites among *Og*, *Osj*, and *Osi*, detecting 218 lineage-specific substitutions. There were 13 introns in 13 genes where substitutions were found at splice sites only in *Osj* (Table 4), suggesting that their ancestral genes were not spliced at these sites. Furthermore, new splice sites were incorporated in the lineage of *Osj* after the divergence between *Osj* and *Osi*. Likewise, nucleotide changes at splice sites of 52 introns of 52 genes were found specifically in the lineage of *Osi*. Although this number was much higher than that in *Osj*, if we examined only splice sites with no substitutions in the flanking sequences (see Experimental procedures), the numbers were 9 and 15 for *Osj* and *Osi*, respectively. Therefore, the apparent excess of the substituted splice sites observed in *Osi* seemed to partially result from misalignments, and the number of substitutions around the splice sites are similar between *Osj* and *Osi*. For 153 introns of 148 genes, *Og* had unique substitutions relative to *Osj* and *Osi*. However, we could not determine whether the *Og* splicing condition was ancestral or derived, due to the lack of an appropriate outgroup.

**Table 4:** Number of introns with lineage-specific donor and/or acceptor site changes

	*Osj*	*Osi*	*Og*
No. of introns^a^	13 (6:7)	52 (25:27)	153 (48:105)
No. of introns between protein-coding exons^b^	7 (6)	33 (27)	109 (107)
No. of introns without stop codons^b^	2 (1)	7 (4)	22 (19)

*Osj*, *Oryza sativa japonica*; *Osi*, *Oryza sativa indica*; *Og*, *Oryza glaberrima*.

a Numbers of donor (left) and acceptor (right) sites are in parentheses.

b Numbers of introns that have GT/AG splicing site motifs are in parentheses.

**Figure 4:**
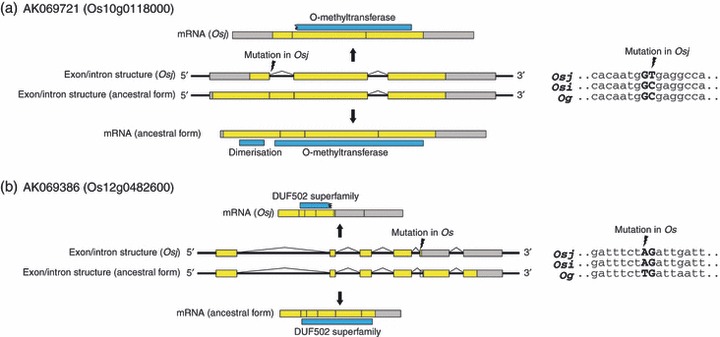
Two examples of *Oryza sativa japonica* (*Osj*) genes that have nucleotide substitutions at splice site motifs. Red arrows on the gene indicate the positions of the substitutions. (a) AK069721 (Os10g0118000), mutation in *Osj* generated a new intron that disrupted the *O*-methyltransferase domain. (b) AK069386 (Os12g0482600), mutation in *Oryza sativa* (*Os*) generated an altered acceptor site that disrupted the DUF502 superfamily domain. Sequence alignments around the splice site motifs are shown on the right. Gray and yellow boxes are untranslated regions and protein-coding regions, respectively. Blue boxes above or below mRNAs indicate functional domains detected by NCBI BLAST searches.

In protein-coding regions, there were a total of 149 introns that had splice sites with lineage-specific substitutions (Table 4). If these were recently created introns, translation of an intron is expected to connect coding frames of the adjacent exons, and a complete coding region will be recovered. In fact, 31 of 149 introns could be translated without stop codons in the protein-coding frames. These results suggest that at least 31 coding regions were recently disrupted by the addition of splice sites. To examine this possibility, we conducted blastp searches of the protein sequences of these 31 genes against the RefSeq database at the ncbi blast server (http://blast.ncbi.nlm.nih.gov/Blast.cgi). As a result, we found that eight proteins lacked parts of functional domains and that these deleted domains were included in the introns. If these possible novel introns were put back in the transcripts, complete functional domains reappeared in four genes (Figure 4). The donor site of the first intron of AK069721 (Os10g0118000) was GT in *Osj*, whereas that in *Osi* and *Og* was GC. Thus, the donor site was probably created in the *Osj* lineage; a blastp search showed that an *O*-methyltransferase domain was truncated at the first splice site, and another dimerization domain appeared in the first exon. In another example of AK069386 (Os12g0482600), the acceptor site of the fourth intron was substituted from TG to AG in both *Osj* and *Osi* after the divergence from *Og* and a conserved but functionally unknown domain was truncated at this junction. In fact, by shifting the acceptor site to 22 bp downstream, we could recover the domain. These observations suggest that creation of novel splice sites disrupted protein-coding genes relatively recently in a specific lineage. This type of drastic change may contribute to differences between species and cultivars. Further investigation of physiological effects derived from genes with altered splice sites may find genes associated with the domestication processes of rice as found in the *waxy* gene. Unfortunately, because our analyses were based on the exon–intron structures of *Osj* genes for which an abundance of full-length cDNAs are available, unique splicing variants that emerged in the *Og* lineage could not be assessed. A comprehensive transcriptome analysis of *Og* would help us further understand the evolution of *Og* genes and find the domestication genes of African rice.

### Evolution of the simple sequence repeats

Our genome survey detected a total of 2451 SSRs that were found in all three rice genomes (see Experimental procedures). The SSRs tended to be densely distributed in genomic regions distant from the centromeric regions (Figure S1). This finding is consistent with the observation that SSR density was correlated with gene density (Hong *et al.*, 2007). These SSRs consisted of 381 di-nucleotide, 1788 tri-nucleotide, and 282 tetra-nucleotide repeats. In particular, the CGC/GCG (13.9%), CGG/CCG (13.5%), and GGC/GCC (9.2%) motifs, which were previously reported to be most abundant in rice genomes (Morgante *et al.*, 2002; Zhang and Xue, 2005), accounted for 50.2% of the tri-nucleotide repeats. Of the 2451 SSRs, 883 were identical among the three genomes, while the remaining 1568 were variable. A comparison of the numbers of polymorphic SSRs between non-transcribed and protein-coding regions revealed that the protein-coding regions contained significantly fewer polymorphic SSRs than the non-transcribed regions (*G*-test, *P* = 3.4 × 10^−15^) (Table 5). This result was anticipated because protein-coding regions are generally under purifying selection, so that sequence changes are less frequent. In addition, previous studies suggested that the excess of tri-nucleotide repeats was attributed to the suppression of other types of repeats whose expansion or contraction may lead to frameshift errors in protein-coding regions (Metzgar *et al.*, 2000; Morgante *et al.*, 2002). In support of this idea, our data showed that 777 of 787 SSRs in protein-coding regions were tri-nucleotide repeats. This proportion was much larger than that (496 of 941) in non-transcribed regions (*G*-test, *P* = 2.2 × 10^−16^).

**Table 5:** Statistics of simple sequence repeats (SSRs)

		Non-transcribed regions			Protein-coding regions		
			
		Di-	Tri-	Tetra-	Di-	Tri-	Tetra-
No. of total SSRs		258	496	187	3	777	7
No. of shared SSRs^a^		16	177	68	0	358	4
No. of polymorphic SSRs		242	319	119	3	419	3
No. of SSRs with lineage-specific length polymorphisms^b^	*Osj*	3	8	11	0	22	0
	*Osi*	26	52	20	0	54	0
	*Og*	74	139	46	1	175	1
No. of SSRs with the same length but lineage-specific polymorphisms^c^	*Osj*	5	9	3	0	23	0
	*Osi*	5	11	6	0	33	1
	*Og*	9	37	17	0	51	0

*Osj*, *Oryza sativa japonica*; *Osi*, *Oryza sativa indica*; *Og*, *Oryza glaberrima*.

a Numbers of SSRs that are identical among the three genomes.

b Numbers of SSRs whose length was different in one of the three genomes and same in the other two.

c Numbers of SSRs whose length was identical among the three genomes but sequence was different in one of the three genomes.

To further examine selection against changes in the length of tri-nucleotide repeats, we counted the number of tri-nucleotide repeats that were different in only one of the three genomes (*Og*, *Osj*, and *Osi*). The number of tri-nucleotide repeats whose length changed and did not change was compared between non-transcribed and protein-coding regions. Although length variations should be deleterious in protein-coding regions, no significant differences were observed in the *Og* and *Osj* lineages between non-transcribed and protein-coding regions. Only *Osi* showed a significantly smaller number of tri-nucleotide repeats in protein-coding regions than in non-transcribed regions (*G*-test, *P* = 0.006). To further examine the evolutionary differences of tri-nucleotide repeats, the number of altered tri-nucleotides repeats between non-transcribed and protein-coding regions among the three rice genomes were subjected to a chi-square test, which did not reveal any significant difference between the genomes. These results imply that, as long as coding frames are preserved, selection against indels is relatively weak in both African and Asian rice species.

The genome size of *Og* was estimated to be smaller than that of *Os* (Martinez *et al.*, 1994; Uozu *et al.*, 1997; Ammiraju *et al.*, 2006). Furthermore, genome sizes vary considerably between *Osj* and *Osi* (International Rice Genome Sequencing Project. 2005, Yu *et al.*, 2005). One possible explanation for the difference in genome size is the lineage-specific accumulation of small changes, which are represented by different SSR lengths. However, the total length (19 099 bp) of SSRs of *Og* in non-exonic regions did not differ significantly from those (19 074 bp and 19 669 bp) of *Osj* and *Osi*, suggesting that the difference in genome size between these species is not caused by the expansion and contraction of SSRs. Therefore, the difference in the genome sizes seems be due to relatively large insertions and deletions.

### Conclusion

In this study, we presented a draft genome sequence of African domesticated rice, *Og*. The assembled contigs correspond to approximately 18% of the *Osj* genome. Additional sequence reads unique to *Og* totaled another 12.7 Mb. Moreover, because SSRs are widely used as genetic markers, the wealth of *Og* SSRs detected in this study should provide breeders with important information for the future development of improved African and Asian cultivars. Further investigation of the *Og* genome sequence and comprehensive and comparative analyses with Asian rice species should help researchers fully explore agronomically useful genes that are preserved in the *Og* genome.

## Experimental Procedures

### Genome sequencing of *Oryza glaberrima*

To enrich gene sequences, two shotgun libraries (with an average fragment size of approximately 2 kb) were prepared by two methods: methylation filtration (Whitelaw *et al.*, 2003) and the subtractive hybridization of repetitive sequences with immobilized and synthesized oligomers designed on the basis of the rice genome sequence.

Target sequences for the subtractive hybridization were widely distributed rice repeats (*copia*1-1, *copia*1-2, *copia*1-3, *copia*2, AF069218, M11585, Retrosat2-1, Retrosat2-2, Retrosat2-3, AF169230, M18203, RCS2) included in TIGR_*Oryza*_Repeats.v2 (Ouyang and Buell, 2004). The PCR primers were designed to amplify entire regions of the repeat elements, and PCR reactions were performed with *O. glaberrima* (IRGC104038) genomic DNA as templates. Biotinylated-UTP was added as one of the nucleotide substrates for the PCR reactions. The resulting biotinylated PCR products were mixed with streptavidin magnetic beads to construct subtraction beads. Three micrograms of *Og* genomic DNA was fragmented by sonication. These sonicated fragments were end-repaired and ligated with adaptors of amplification primers. The double-stranded genomic fragments were heat-denatured and mixed with the subtraction beads. The pass-through fractions were ethanol-precipitated, and single-stranded fragments were converted to double-stranded (ds) DNA by amplification with PCR primers designed from the adaptor regions. Amplified ds-fragments were ligated with pGEM-T easy vector (Promega, http://www.promega.com/) using T-overhang structures of the fragment. *Escherichia coli* DH10B cells were transformed with the ligated construct to construct a subtraction library.

Each library constructed in the previous step contained about 125 000 subclones. We thereafter sequenced all of these subclones from both ends by using the capillary sequencer ABI3700, which initially yielded a total of 220.3 Mb genomic sequences.

### Construction of genome alignments with *Oryza sativa*

For each shotgun read, we trimmed low-quality nucleotides from the 3′-end as long as the phred score was <15. Sequences of <100 bp in length were discarded. We also discarded the sequences that showed similarities to the organelle genomes with nucleotide identities of ≥95%, *E*-value of ≤1.0 × 10^−10^, and sequence coverage of ≥90%. As a result, we obtained a total of around 206 Mb from 437 642 sequence reads (Table 1, Figure S2). We further masked repetitive regions in the shotgun sequence reads in lower case using RepeatMasker (http://www.repeatmasker.org/) with the MIPS Repeat Element Database (mips-REdat) (Spannagl *et al.*, 2007). Sequence reads with short non-repetitive nucleotides (<30 bp) were excluded from our analyses. The remaining shotgun sequences were mapped to the genome sequences of *Osj* (IRGSP build 4) (International Rice Genome Sequencing Project, 2005) and *Osi* (Yu *et al.*, 2005). BLASTN (Altschul *et al.*, 1997) was used with the nucleotide identity threshold set to ≥90% and an *E*-value of ≤1.0 × 10^−10^ for mapping. The *Osj* genome sequence was downloaded from the RAP-DB (Tanaka *et al.*, 2008), and the *Osi* genome sequence was downloaded from DDBJ/EMBL/GenBank (accession numbers CM000126–CM000137). If a shotgun sequence was mapped to multiple positions, the position with the highest nucleotide identity, lowest *E*-value, and highest score was selected. A genome-wide alignment between *Osj* and *Og* was constructed by concatenating the mapped reads. If more than one *Og* sequence overlapped on the *Osj* genome and the sequences differed, the *Og* nucleotide with the highest phred score was selected for the position. An *Og* nucleotide obtained from a sequence selected by the methylation–filtration method was preferentially chosen if two or more nucleotides had the same phred score. The *Osi* genome sequence was aligned with the *Osj* genome using BLASTZ with the following parameters: *C* = 0, *H* = 2000, *Y* = 3400, and *T* = 4 (Schwartz *et al.*, 2003; Matsuya *et al.*, 2008).

### Functional classification of *Oryza glaberrima* genes

To investigate the types of genes that reside in the *Og*-specific genomic portions, we first assembled 111 736 unmapped shotgun sequences by the CAP3 program using the default settings (Huang and Madan, 1999). This method yielded 4598 contigs and 20 461 singletons. Second, we conducted BLASTN searches using the contigs and singletons as queries against the organelle genomes and excluded sequences that showed ≥90% nucleotide identities. Finally, protein-coding regions were inferred in these contigs and singletons on the basis of sequence similarity. Protein sequences deposited in the Swiss-Prot database (The UniProt Consortium 2009) were aligned with *Og*-specific sequences by ProSplign (http://www.ncbi.nlm.nih.gov/sutils/static/prosplign/prosplign.html). If positions of alignments overlapped on a sequence, they were clustered and regarded as a single possible locus, and one representative alignment was selected on the basis of amino acid identity and coverage. If more than one protein sequence showed the same identity and coverage, a sequence with start and stop codons and without frame disruptions in the alignments was selected. Alignments based on proteins of bacteria, viruses, or transposable elements were discarded. As a result, we obtained 1277 loci inferred from 1002 proteins on 1266 *Og*-specific sequences (260 contigs and 1006 singletons). Translated amino acid sequences of these 1277 loci were subjected to InterProScan searches (Quevillon *et al.*, 2005). We excluded the sequences with transposable element-associated InterPro domains. Following the Gene Ontology (GO) hierarchy, the functions were categorized by the map2slim program with generic GO slims.

### Analysis of amino acid substitutions

Pairwise codon alignments of genes between *Osj* and *Og* were constructed on the basis of the positions of the protein-coding regions determined by the genome annotation of *Osj* (Tanaka *et al.*, 2008). To avoid sequencing errors of *Og*, we selected only nucleotides supported by two or more shotgun reads with the phred score of ≥20, and if different nucleotides were seen at the same site, one nucleotide that was in the majority was used. Orthologous gene pairs between *Osj* and sorghum were assigned by the mutual best-hit strategy after conducting BLASTP searches of *Osj* and sorghum protein sequences (Tanaka *et al.*, 2008; Paterson *et al.*, 2009). Codon alignments based on the protein alignments were constructed using PAL2NAL (Suyama *et al.*, 2006). If *Og* or sorghum sequences contained gaps and/or premature stop codons, the alignment was discarded. The two pairwise alignment sets between *Osj* and *Og* and between *Osj* and sorghum were integrated into multiple alignments of the three species. As a result, 7656 alignments with 100 or more amino acids or over 70% coverage of RAP proteins were selected and used for further analyses. For amino acid substitution analysis, to remove ambiguity of misalignments in the terminal regions, the 10 amino acids at the N- and C-termini were not used. Synonymous and nonsynonymous distances were calculated by the modified Nei–Gojobori method (Zhang *et al.*, 1998), and the branch lengths leading to *Osj* and *Og* were estimated by the least squares method. Standard deviations of the ratio of nonsynonymous to synonymous distances were calculated by the bootstrap test with 1000 iterations.

### Analysis of nucleotide substitutions at splice sites

We compared the sequences of the splice sites and their flanking regions in the *Og* genome with those in the *Osj* and *Osi* genomes. A splice site was used for our analysis if the site and surrounding 10-bp in the 5′- and 3′-flanking regions of both *Og* and *Osi* genomes were aligned with the *Osj* genome without gaps. If substitutions were observed in splice sites that were located in protein-coding regions, we assessed the coding potentials of the introns by checking for stop codons in the possible coding frames of the ‘introns’.

### Simple sequence repeat marker polymorphism

The SSRs with minimal repeat units of nine, six, and five for di-, tri-, and tetra-nucleotide SSRs, respectively, were detected in each of the *Osj*, *Osi*, and *Og* genomes by the SSRIT program (Temnykh *et al.*, 2001). Positions of the SSRs on the *Osi* and *Og* genomes were converted to those on the *Osj* genome, and all SSRs were merged and clustered on the *Osj* genome. To exclude ambiguity of repetitive sequences, we selected SSRs that did not contain any gaps within the 50-bp upstream and downstream regions.

## SUPPORTING INFORMATION

**Figure S1.** An overview of the simple sequencerepeats (SSRs) of the *Oryza sativa japonica* (*Osj*)genome.

**Figure S2.** Workflow of sequence processing, mapping, and assembling.

**Table S1.** Numbers of nucleotides that matchedtarget repeats employed in our subtractive hybridization. Resultsof repeat-masking in the subset used in this study for thesubtractive hybridization were compared with those of bacterialartificial chromosome (BAC) end sequences of a previous study(Ammiraju *et al.*, 2006).

**Table S2.** Numbers of lineage-specific amino acid substitutions with and without property changes based on four classification categories.

**Table S3.** Numbers of synonymous(*d*_s_) and nonsynonymous (*d*_n_)substitutions and their ratio(*d*_n_/*d*_s_) in the lineages of*Oryza sativa japonica* (*Osj*) and *Oryzaglaberrima* (*Og*).

As a service to our authors and readers, this journal provides supporting information supplied by the authors. Such materials are peer-reviewed and may be re-organized for online delivery, but are not copy-edited or typeset. Technical support issues arising from supporting information (other than missing files) should be addressed to the authors.
