# Supplementary figures and images for: Distinct evolutionary patterns of Oryza glaberrima deciphered by genome sequencing and comparative analysis
Source: Plant J. 2011 Mar 21;66(5):796–805. doi: 10.1111/j.1365-313X.2011.04539.x (PMC3568898; doi:10.1111/j.1365-313X.2011.04539.x)

Figure S1

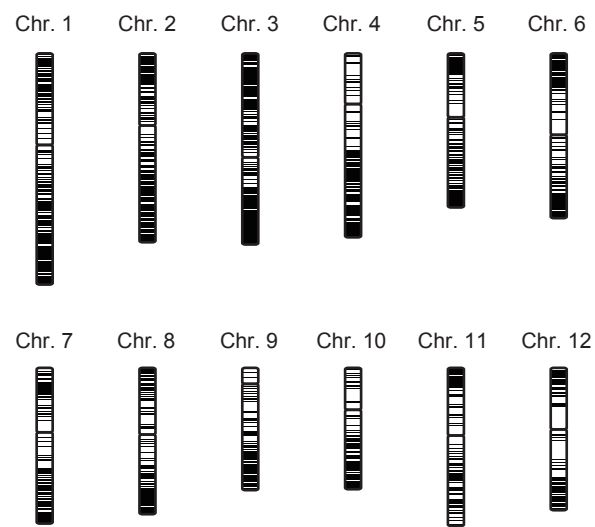

Supplement: Supplementary file 1 [file tpj0066-0796-SD1.pdf]

Figure S2

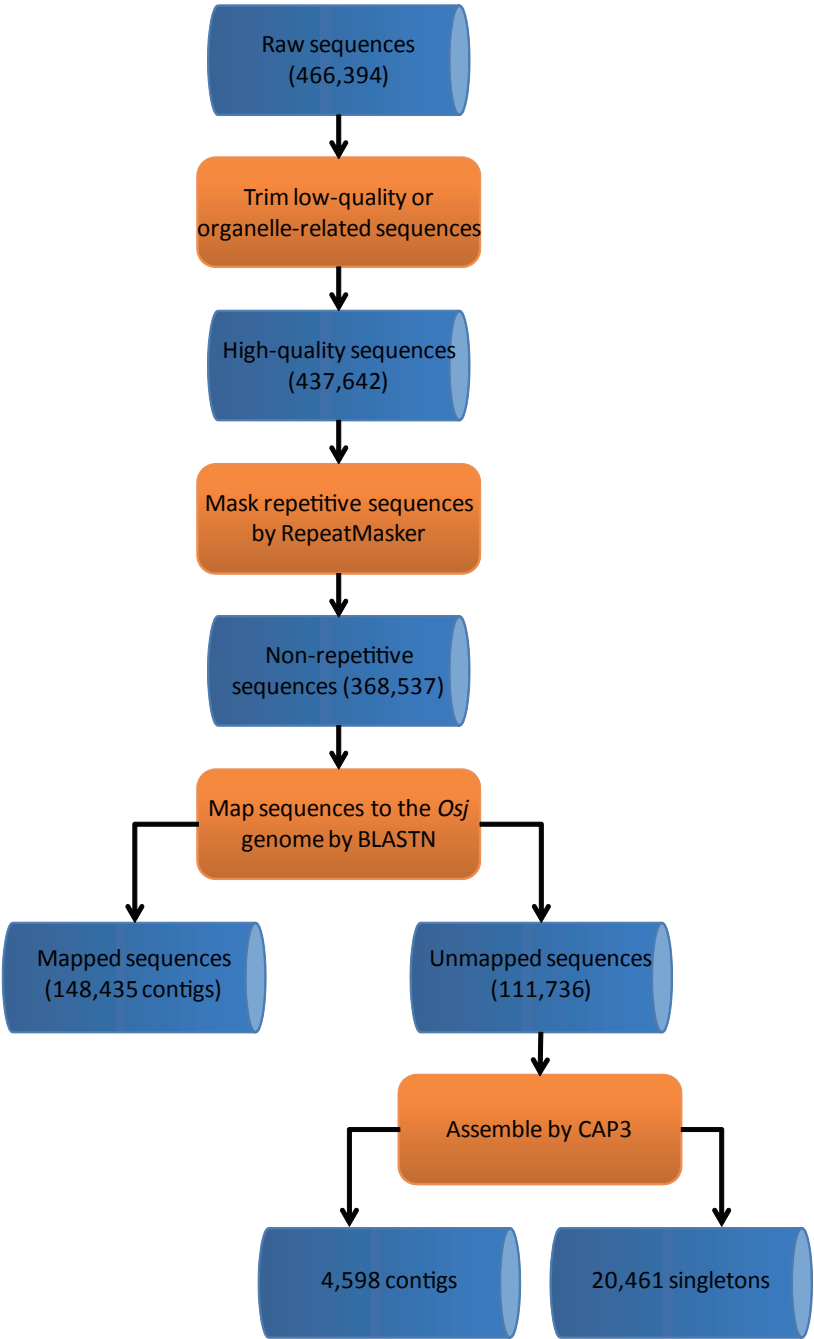

Supplement: Supplementary file 2 [file tpj0066-0796-SD2.pdf]
