# Supplementary material for: Distinct evolutionary patterns of Oryza glaberrima deciphered by genome sequencing and comparative analysis
Source: Plant J. 2011 Mar 21;66(5):796–805. doi: 10.1111/j.1365-313X.2011.04539.x (PMC3568898; doi:10.1111/j.1365-313X.2011.04539.x)
Supplement: Supplementary file 3 [file tpj0066-0796-SD3.doc]

**Supporting Information**

**Figure S1:** An overview of the simple sequence repeats (SSRs) of the *Osj* genome.

**Figure S2:** Workflow of sequence processing, mapping, and assembling.

**Table S1:** Numbers of nucleotides that matched target repeats employed in our subtractive hybridization. Results of repeat-masking in the subset used in this study for the subtractive hybridization were compared with those of bacterial artificial chromosome (BAC) end sequences of a previous study (Ammiraju*, et al.* 2006).

**Table S2:** Numbers of lineage-specific amino acid substitutions with and without property changes based on four classification categories.

**Table S3:** Numbers of synonymous and nonsynonymous substitutions and their ratio (*d*_n_/*d*_s_) in the lineages of *Osj* and *Og*.
