# Supplementary material for: Distinct evolutionary patterns of Oryza glaberrima deciphered by genome sequencing and comparative analysis
Source: Plant J. 2011 Mar 21;66(5):796–805. doi: 10.1111/j.1365-313X.2011.04539.x (PMC3568898; doi:10.1111/j.1365-313X.2011.04539.x)
Supplement: Supplementary file 4 [file tpj0066-0796-SD4.doc]

**Table S1:** Numbers of nucleotides that matched target repeats employed in our subtractive hybridization. Results of repeat-masking in the subset used in this study for the subtractive hybridization were compared with those of bacterial artificial chromosome (BAC) end sequences of a previous study (Ammiraju*, et al.* 2006).

|  | Targeted repetitive elements (bp) | Non-targeted sequences (bp) |
| --- | --- | --- |
| Subtractive hybridization | 435,595 | 98,461,856 |
| BAC end sequences | 280,249 | 37,488,915 |

**Table S2:** Numbers of lineage-specific amino acid substitutions with and without property changes based on four classification categories.

Category (A) by the maximum correlation with the *d*_N_/*d*_S_ ratio

|  | No property change | Property change |
| --- | --- | --- |
| *Osj* | 183 | 132 (52:80) |
| *Og* | 163 | 123 (50:73) |

Category (B) by polarity and volume

|  | No property change | Property change |
| --- | --- | --- |
| *Osj* | 160 | 155 (68:87) |
| *Og* | 149 | 137 (60:77) |

Category (C) by charge and aromatic

|  | No property change | Property change |
| --- | --- | --- |
| *Osj* | 212 | 103 (42:61) |
| *Og* | 193 | 93 (46:47) |

Category (D) by charge and polarity

|  | No property change | Property change |
| --- | --- | --- |
| *Osj* | 162 | 153 (62:91) |
| *Og* | 140 | 146 (71:75) |

Note. - Numbers in parentheses show numbers of property changes inside (left) and outside (right) Pfam domains.

**Table S3:** Numbers of synonymous and nonsynonymous substitutions and their ratio (*d*_n_/*d*_s_) in the lineages of *Osj* and *Og*.

|  | Total no. of synonymous substitutions | No. of synonymous substitutions per site (*d*_s_) | Total no. of nonsynonymous substitutions | No. of nonsynonymous substitutions per site (*d*_n_) | *d*_n_/*d*_s_^b^ |
| --- | --- | --- | --- | --- | --- |
| CA^a^ - *Oj* | 1372 | 0.00471 | 1102 | 0.00116 | 0.24683 (0.01310) |
| CA^a^ - *Og* | 1276 | 0.00405 | 1148 | 0.00122 | 0.30092 (0.01681) |

^a^CA: Common ancestor of *Osj* and *Og*.

^b^Standard deviations of the ratios of nonsynonymous to synonymous distances are in parentheses.
